# A Novel Model Combining Tumor Length, Tumor Thickness, TNM_Stage, Nutritional Index, and Inflammatory Index Might Be Superior to the 8th TNM Staging Criteria in Predicting the Prognosis of Esophageal Squamous Cell Carcinoma Patients Treated With Definitive Chemoradiotherapy

**DOI:** 10.3389/fonc.2022.896788

**Published:** 2022-06-01

**Authors:** Xiaohui Chen, Yilin Yu, Haishan Wu, Jianjian Qiu, Dongmei Ke, Yahua Wu, Mingqiang Lin, Tianxiu Liu, Qunhao Zheng, Hongying Zheng, Jun Yang, Zhiping Wang, Hui Li, Lingyun Liu, Qiwei Yao, Jiancheng Li, Wenfang Cheng

**Affiliations:** ^1^ Department of Thoracic Surgery, Fujian Medical University Cancer Hospital, Fujian Cancer Hospital, Fuzhou, China; ^2^ College of Clinical Medicine for Oncology, Fujian Medical University, Fuzhou, China; ^3^ Graduate School of Fujian Medical University , Fuzhou, China; ^4^ Department of Radiation Oncology, Fujian Medical University Cancer Hospital, Fujian Cancer Hospital, Fuzhou, China

**Keywords:** novel prognostic model, tumor length, tumor thickness, esophageal squamous cell carcinoma, prognostic index score

## Abstract

**Background:**

We aimed to determine whether the tumor length and tumor thickness should be used as prognostic factors for esophageal squamous cell carcinoma (ESCC) patients treated with definitive chemoradiotherapy (dCRT).

**Methods:**

A retrospective analysis consists of 902 non-operative ESCC patients received dCRT. The nomogram was used to predict the survival. Besides, Restricted Cubic Splines (RCS) was used to examine the relationship between prognostic factors and survival outcomes. Finally, the prognostic index (PI) scores were constructed according to the tumor length and tumor thickness, and the patients were divided into the low-, medium-, and high-risk groups.

**Results:**

The median follow-up of overall survival (OS) and progression-free survival (PFS) were 23.0 months and 17.5 months. Multivariate Cox regression analysis showed that tumor length and tumor thickness were independent prognostic factors associated with survival. Our novel nomograms for OS and PFS were superior to the TNM classification (p < 0.001). Besides, RCS analysis demonstrated that the death hazard of tumor length and tumor thickness sharply increased at 7.7 cm and 1.6 cm (p < 0.001). Finally, there were significant differences for ESCC patients with clinical TNM stage group of the OS and PFS in different risk groups. The higher risk group was significantly associated with shorter OS and PFS in ESCC patients (both p < 0.001 for all).

**Conclusion:**

The study results suggest that the novel models integrating tumor length and tumor thickness may provide a simple and widely available method for evaluating the prognosis of non-operative ESCC patients. The tumor length and tumor thickness should be considered as prognostic factors for ESCC.

## Introduction

Esophageal carcinoma (EC) ranks seventh and sixth in the world in morbidity and total mortality, respectively. It affects more than 550,000 people worldwide, and its incidence is on the rise (1, 2). There are two main types of EC: esophageal squamous cell carcinoma (ESCC) and esophageal adenocarcinoma (EAD). Although the treatment of EC has been continuously updated and improved in recent years, the 5-year survival rate is still less than 20% (3). For inoperable patients, radiotherapy, chemotherapy, targeted therapy, and immunotherapy are the treatment options (4). However, among these patients, the TNM classification mainly depends on modern imaging techniques. Due to individual differences, the survival rates of patients with the same clinical TNM stage may differ even after undergoing similar treatments. The guiding significance and predictive value for patients receiving non-operative treatment are limited by the clinical TNM classification (5, 6). Therefore, it is reasonable to develop a more practical and feasible prognostic model for EC, especially for patients who have received definitive chemoradiotherapy (dCRT).

For the diagnosis of EC, tumor length was excluded from the TNM staging. However, increasing evidence shows that the tumor length affects the prognosis of EC patients (7, 8). Moreover, some studies have shown that tumor thickness is also related to the prognosis of EC (9, 10). Some protein markers or genes have also been reported to predict the survival of patients with EC (11, 12), but the acquisition of these indicators is complicated and time-consuming. Therefore, it is imperative to identify readily available markers that can predict patient survival. Inflammatory indices have been demonstrated to be associated with the prognosis of some cancers, including ESCC (13–16). However, controversy exists about which is the best inflammatory index for prognostic prediction in EC. Besides, the prognosis of cancer patients depends not only on the tumor but also on the host factors. As is known to all, the condition of nutrition significantly affected the prognosis of EC patients.

Recently, many scholars have investigated the use of reliable prognostic factors to predict the prognosis of EC patients (17). Unfortunately, there is no accurate method to reliably predict the prognosis of EC patients undergoing non-surgical treatment. Therefore, it is urgent to determine which patients have a higher risk of death after treatment to better tailor the treatment strategies based on the risk of death. In this study, we established a new prognostic model that combines tumor length, thickness, N stage, TNM stage, inflammatory indices, and nutritional indices. To the best of our knowledge, there is no study about the comparation between the TNM classification and other models on the prognostic evaluation of esophageal squamous cell carcinoma (ESCC) treated with definitive chemoradiotherapy (dCRT). The purpose of this study was to determine whether this model is superior to predict the prognosis of patients with ESCC received dCRT than the 8th TNM classification alone and whether the tumor length and tumor thickness should be used as prognostic factors for esophageal squamous cell carcinoma.

## Methods

### Patient Eligibility

The eligible cases included 902 patients diagnosed with ESCC at Fujian Cancer Hospital between January 2011 and December 2020. The inclusion criteria were as follows: (A) histologically confirmed ESCC; (B) not previously treated; (C) Karnofsky score ≥ 70 points; (D) radiotherapy dose 50–70 Gy (25-35 fractions in 5-7 weeks), 0-9 courses of platinum-based chemotherapy, (E) no distant metastasis, and (F) no other major diseases. Clinical staging was performed according to the 8th edition of the TNM staging criteria. Blood biochemical data were collected within one week before commencing therapy. In our hospital, patients need to carry out routine blood tests before treatment. In our study, all patients’ blood biochemical information was obtained on any of the one week prior to treatment. In addition, blood information obtained more than one week before treatment or at any time after treatment will be excluded. Tumor length was determined by barium esophagography, and tumor thickness was measured using computed tomography (CT). In 2010, the Chinese clinical staging expert group proposed that the tumor length determined by barium esophagography and the tumor diameter determined by the maximum esophageal diameter shown by CT be considered criteria for the nonsurgical T staging of esophageal cancer. Besides, patients who were unable to undergo a barium swallowing test due to severe dysphagia were excluded in our research. The clinical T and N stages of all patients were comprehensively analyzed by at least two experienced clinicians based on all the examination results of the patients. The clinical staging was performed according to the 8th edition of the TNM staging criteria based on all the examination results. The study was conducted in accordance with the principles of the Declaration of Helsinki and was approved by the ethics committee of the Fujian Cancer Hospital (YKT2021-005-01).

### Definitive Chemoradiotherapy

Radiotherapy: All patients received external irradiation with a linear accelerator. Radiotherapy was performed in the form of intensity modulated radiotherapy (IMRT) or three dimensional-conformal radiotherapy (3D-CRT). Before delineating the tumor target volume, imaging information obtained from barium esophagography, endoscopic examination, endoscopic ultrasound (EUS), CT, or positron emission tomography-CT (PET-CT) scan was analyzed in detail. We treated the tumors and metastatic lymph nodes with a total dose of 50-70 Gy in 25-35 fractions over 5-7 weeks. A 95% isodose line was required for the prescription dose. Additional parameters included: V20 ≤ 30%, V5 ≤ 65%, average mean lung dose (MLD) ≤ 18 Gy in both lungs, V40 ≤ 40% in the heart, and Dmax ≤ 45 Gy in the spinal cord. The gross tumor volume (GTV) was based on the esophageal tumors seen on CT imaging (tumor length was based on barium esophagography, endoscopy, and other imaging techniques). Based on the GTV, the clinical target volume (CTV) was expanded by 5 mm in the anterior and posterior directions as well as the left and right directions, and at least 30 mm in the cranial and caudal direction. Based on the GTV and CTV, the planning target volume (PTV) was expanded by 5 mm in the anterior, posterior, left, and right directions, and 10 mm in the cranial and caudal direction.

Chemotherapy: Patients received simultaneous and/or sequential chemotherapy, and the treatment course was divided into 0-9 courses. The chemotherapy regimen was based on platinum, including (A) paclitaxel d1 + nedaplatin d2 or cisplatin d2 or lobaplatin d2 or carboplatin AUC2 d2; (B) 5-fluorouracil (5-FU) d1-2 + cisplatin d2.

### Definition of Nutritional Index and Inflammatory Index

The weight divided by the square of height to calculate the body mass index (BMI). The serum albumin level + 5 multiplied by the absolute lymphocytes count to calculate the prognostic nutrition index (PNI). The absolute number of lymphocytes divided by the absolute number of monocytes was used to calculate the lymphocyte-to-monocyte ratio (LMR). The absolute number of neutrophils was divided by the absolute number of lymphocytes to calculate the neutrophil-to-lymphocyte ratio (NLR). The absolute number of platelets divided by the absolute number of lymphocytes was used to calculate the platelet-to-lymphocyte ratio (PLR).

### The Prognostic Index Score

The prognostic index (PI) score was made up by the combination of the tumor length and tumor thickness. The best cut-off values of tumor length and tumor thickness were 7.7cm and 1.6cm. In general, patients with both increased tumor length (≥7.7cm) and tumor thickness (≥1.6cm) were assigned to a score of 3. Patients with one increased tumor length (≥7.7cm) or tumor thickness (≥1.6cm) were assigned to 2. Patients with no increased tumor length (≥7.7cm) and tumor thickness (≥1.6cm) were assigned to 1. Therefore, we classified the PI score into three groups [PS (Prognostic score) = 1, 2, and 3, respectively]. PS = 1, 2, and 3 mean low-risk, medium-risk, and high-risk group, respectively.

### Evaluation Strategy and Follow-Up

The study endpoints were overall survival (OS) and progression-free survival (PFS). OS was defined as the period from the pathological diagnosis to death or last follow-up. PFS was defined as the period from the pathological diagnosis to tumor progression, death, or the last follow-up. A follow-up evaluation was conducted every three months in the first year, every six months in the following two years, and once a year thereafter until the end of the study. During the follow-up period, the patients were examined regularly. The assessments included physical examination, routine blood tests, biochemistry, tumor markers, late radiotoxicity assessment, barium esophagography, chest/abdominal CT scan, endoscopy, and PET-CT. Follow-up information was obtained from the patients’ medical records and/or telephone interviews. April 2021 was the final censoring date for assessing the survival time.

### Statistical Analysis

All statistical analyses were performed using the SPSS (version 26.0) and R (version 4.0.2) software. The optimal cutoff values for RT dose, tumor length, tumor thickness, PNI, BMI, LMR, NLR, and PLR were calculated using the X-tile application (https://medicine.yale.edu/lab/rimm/research/software/). The survival curve was drawn using the Kaplan-Meier method. The Cox regression model was used for univariate and multivariate analyses. All factors with p < 0.05 in the univariate analysis were included in the multivariate analysis to determine the independent prognostic factors for ESCC. The rms R package was used to generate a nomogram. The C-index and calibration curves were obtained using the timeROC R package and the Hmisc R package, respectively. In this study, the C-index and calibration curves were used to determine the nomogram’s discrimination and calibration ability, respectively. The Delong’s test was used to compare our prediction model and the 8th TNM staging criteria in the prediction of 1-, 3-, and 5-year OS and PFS. Finally, Restricted Cubic Splines (RCS) was used to examine the relationship between factors and survival outcomes by the rms R package. All analyses were bilateral, and p-values less than 0.05 were considered statistically significant.

## Results

### Clinical Characteristics

As shown in **Table 1**, 902 eligible patients with ESCC participated in the study. Data on patient characteristics, including clinical features (gender, age, weight loss, RT dose, tumor location, tumor length, tumor thickness, and tumor stage), nutritional and inflammatory indices (PNI, BMI, LMR, NLR, and PLR) were collected. A total of 649 men and 253 women were included in our study. According to the TNM stage, T2 was found in 57 patients (6.3%), T3 in 443 (49.1%), and T4 in 402 (44.6%). N0 was found in 264 patients (29.3%), N1 in 392 (43.5%), N2 in 192 (21.3%), and N3 in 54 (6.0%). The clinical stage distribution included 21.8% stage II patients (n = 197), 29.8% stage III (n = 269), and 48.3% stage IV (n = 436). The optimal cutoff values for RT dose, tumor length, tumor thickness, PNI, BMI, LMR, NLR, and PLR were calculated to be 59.9Gy, 7.7cm, 1.6cm, 41.7, 19.7, 3.26, 4.57, and 180.56, respectively. A total of 547 patients (60.6%) died, while 355 patients (39.4%) were alive.

**Table 1:** Clinical characteristics of 902 ESCC patients.

Clinicopathologic variable		Total(N)	Percentage (%)
Gender			
	Male	649	72.0%
	Female	253	28.0%
Age (year)			
	<65	496	55.0%
	≥65	406	45.0%
Weight loss			
	Yes	438	48.6%
	No	464	51.4%
RT dose (Gy)			
	<59.9	155	17.2%
	≥59.9	747	82.8%
Tumor location			
	Cervical	89	9.9%
	Upper thoracic	248	27.5%
	Middle thoracic	92	10.2%
	Lower thoracic	473	52.4%
Tumor length (cm)			
	<7.7	152	16.9%
	≥7.7	750	83.1%
Tumor thickness (cm)			
	<1.6	348	38.6%
	≥1.6	554	61.4%
T stage			
	T2	57	6.3%
	T3	443	49.1%
	T4	402	44.6%
N stage			
	N0	264	29.3%
	N1	392	43.5%
	N2	192	21.3%
	N3	54	6.0%
TNM stage			
	Stage II	197	21.8%
	Stage III	269	29.8%
	Stage IV	436	48.3%
PNI			
	<41.7	119	13.2%
	≥41.7	783	86.8%
BMI			
	<19.7	265	29.4%
	≥19.7	637	70.6%
LMR			
	<3.26	291	32.3%
	≥3.26	611	67.7%
NLR			
	<4.57	803	89.0%
	≥4.57	99	11.0%
PLR			
	<180.56	696	77.2%
	≥180.56	206	22.8%

ESCC, esophageal squamous cell carcinoma; T, tumor; N, node; TNM, tumor-node-metastasis; LMR, lymphocyte-to-monocyte ratio; NLR, neutrophil-to-lymphocyte ratio; PLR, platelet-to-lymphocyte ratio.

### Univariate and Multivariate Survival Analyses for OS in ESCC

The median follow-up period of OS was 23.0 months (2.1 to 124.7 months). Univariate and multivariate Cox regression models for predictors of OS are shown in **Table 2**. Univariate analyses demonstrated that RT dose (p < 0.001), tumor location (p = 0.002), tumor length (p < 0.001), tumor thickness (p < 0.001), T stage (p = 0.003), N stage (p < 0.001), TNM stage (p < 0.001), PNI (p < 0.001), BMI (p < 0.001), LMR (p < 0.001), NLR (p < 0.001), and PLR (p < 0.001) were the significant risk factors for a worse OS. On multivariate analysis, the tumor length (p < 0.001; hazard ratio [HR], 1.685; 95% confidence interval [CI], 1.362–2.085), tumor thickness (p < 0.001; HR, 1.514; 95% CI, 1.263–1.815), N stage (p < 0.001; HR, 1.520; 95% CI, 1.247–1.851), TNM stage (p = 0.044; HR, 1.331; 95% CI, 1.008–1.756), PNI (p = 0.004; HR, 1.475; 95% CI, 1.129–1.927), BMI (p < 0.001; HR, 1.509; 95% CI, 1.253–1.817), and LMR (p = 0.021; HR, 1.251; 95% CI, 1.035–1.511) were independently associated with a worse OS. The 1-,3-, and 5-year OS rates were 84.7%, 47.9%, 37.5% and 63.8%, 20.8%, 9.6% for a tumor length < 7.7 cm and tumor length ≥ 7.7 cm, respectively. Besides, the 1-,3-, and 5-year OS rates were 85.7%, 52.2%, 44.0% and 74.0%, 29.9%, 16.2% for a tumor thickness < 1.6 cm and tumor thickness ≥ 1.6 cm, respectively. The results showed that OS was significantly correlated with the tumor length and tumor thickness in ESCC patients.

**Table 2:** Univariate and multivariate analyses of prognostic factors for OS in patients with ESCC.

Clinicopathologic parameters	Univariate analysis				Multivariate analysis		
	HR	95% CI	p		HR	95% CI	p
Gender							
Male vs. Female	1.163	0.959-1.410	0.124				
Age (years)							
≥65 vs. <65	1.028	0.868-1.216	0.750				
Weight loss							
Yes vs. No	1.125	0.951–1.330	0.169				
RT dose (Gy)							
<59.9 vs. ≥59.9	1.480	1.194-1.833	<0.001		1.222	0.981-1.522	0.074
Tumor location							
Cervical/Upper vs. Middle/Lower	0.755	0.631-0.902	0.002		0.846	0.705-1.015	0.072
Tumor length (cm)							
≥7.7 vs. <7.7	2.463	2.021-3.002	<0.001		1.685	1.362-2.085	<0.001
Tumor thickness (cm)							
≥1.6 vs. <1.6	1.967	1.662-2.327	<0.001		1.514	1.263-1.815	<0.001
T stage							
T4 vs. T2/T3	1.290	1.090-1.526	0.003		1.003	0.827-1.217	0.973
N stage							
N2/N3 vs. N0/N1	1.928	1.613-2.306	<0.001		1.520	1.247-1.851	<0.001
TNM stage							
Stage III/IV vs. Stage II	1.898	1.506-2.394	<0.001		1.331	1.008-1.756	0.044
PNI							
<41.7 vs. ≥41.7	2.040	1.630-2.553	<0.001		1.475	1.129-1.927	0.004
BMI							
<19.7 vs. ≥19.7	1.844	1.549-2.196	<0.001		1.509	1.253-1.817	<0.001
LMR							
<3.26 vs. ≥3.26	1.577	1.327-1.874	<0.001		1.251	1.035-1.511	0.021
NLR							
≥4.57 vs. <4.57	1.874	1.472-2.385	<0.001		1.092	0.823-1.450	0.540
PLR							
≥180.56 vs. <180.56	1.630	1.349-1.969	<0.001		1.083	0.865-1.355	0.486

OS, overall survival; ESCC, esophageal squamous cell carcinoma; HR, hazard ratio; 95% CI, 95% confidence interval; T, tumor; N, node; TNM, tumor-node-metastasis; LMR, lymphocyte-to-monocyte ratio; NLR, neutrophil-to-lymphocyte ratio; PLR, platelet-to-lymphocyte ratio.

### Univariate and Multivariate Survival Analyses for PFS in ESCC

The median follow-up period of PFS was 17.5 months (1.1 to 124.7 months). Univariate and multivariate Cox regression models for predictors of PFS are shown in **Table 3**. Univariate analyses also demonstrated that the RT dose (p = 0.005), tumor location (p = 0.007), tumor length (p < 0.001), tumor thickness (p < 0.001), T stage (p = 0.001), N stage (p < 0.001), TNM stage (p < 0.001), PNI (p < 0.001), BMI (p < 0.001), LMR (p < 0.001), NLR (p < 0.001), and PLR (p < 0.001) were the significant risk factors for a worse PFS. Multivariate analysis showed that the tumor length (p < 0.001; hazard ratio [HR], 1.750; 95% confidence interval [CI], 1.419–2.158), tumor thickness (p < 0.001; HR, 1.510; 95% CI, 1.264–1.805), N stage (p < 0.001; HR, 1.502; 95% CI, 1.239–1.820), TNM stage (p = 0.018; HR, 1.384; 95% CI, 1.056–1.814), PNI (p = 0.004; HR, 1.461; 95% CI, 1.130–1.889), BMI (p < 0.001; HR, 1.471; 95% CI, 1.226–1.765), and LMR (p = 0.020; HR, 1.247; 95% CI, 1.036–1.500) were independently associated with a worse PFS. The results revealed that PFS was significantly correlated with the tumor length and tumor thickness in patients with ESCC. The 1-,3-, and 5-year PFS rates were 71.0%, 43.3%, 35.5% and 43.4%, 16.9%, 7.1% for a tumor length < 7.7 cm and tumor length ≥ 7.7 cm, respectively. In addition, the 1-,3-, and 5-year PFS rates were 72.7%, 48.6%, 40.7% and 56.2%, 23.9%, 15.3% for a tumor thickness < 1.6 cm and tumor thickness ≥ 1.6 cm, respectively.

**Table 3:** Univariate and multivariate analyses of prognostic factors for PFS in patients with ESCC.

Clinicopathologic parameters	Univariate analysis				Multivariate analysis		
	HR	95% CI	p		HR	95% CI	p
Gender							
Male vs. Female	1.175	0.974-1.418	0.092				
Age (years)							
≥65 vs. <65	0.985	0.836-1.161	0.857				
Weight loss							
Yes vs. No	1.162	0.986–1.368	0.073				
RT dose (Gy)							
<59.9 vs. ≥59.9	1.359	1.100-1.679	0.005		1.120	0.901-1.392	0.307
Tumor location							
Cervical/Upper vs. Middle/Lower	0.786	0.661-0.935	0.007		0.886	0.742-1.057	0.179
Tumor length (cm)							
≥7.7 vs. <7.7	2.538	2.090-3.083	<0.001		1.750	1.419-2.158	<0.001
Tumor thickness (cm)							
≥1.6 vs. <1.6	1.967	1.669-2.319	<0.001		1.510	1.264-1.805	<0.001
T stage							
T4 vs. T2/T3	1.333	1.131-1.570	0.001		1.013	0.839-1.223	0.891
N stage							
N2/N3 vs. N0/N1	1.887	1.584-2.248	<0.001		1.502	1.239-1.820	<0.001
TNM stage							
Stage III/IV vs. Stage II	1.969	1.570-2.469	<0.001		1.384	1.056-1.814	0.018
PNI							
<41.7 vs. ≥41.7	1.973	1.583-2.460	<0.001		1.461	1.130-1.889	0.004
BMI							
<19.7 vs. ≥19.7	1.778	1.498-2.110	<0.001		1.471	1.226-1.765	<0.001
LMR							
<3.26 vs. ≥3.26	1.545	1.304-1.829	<0.001		1.247	1.036-1.500	0.020
NLR							
≥4.57 vs. <4.57	1.807	1.423-2.294	<0.001		1.078	0.818-1.421	0.592
PLR							
≥180.56 vs. <180.56	1.605	1.334-1.930	<0.001		1.115	0.899-1.384	0.321

PFS, progression-free survival; ESCC, esophageal squamous cell carcinoma; HR, hazard ratio; 95% CI, 95% confidence interval; T, tumor; N, node; TNM, tumor-node-metastasis; LMR, lymphocyte-to-monocyte ratio; NLR, neutrophil-to-lymphocyte ratio; PLR, platelet-to-lymphocyte ratio.

### Establishment of Prognostic Model for ESCC

**Figure 1:**
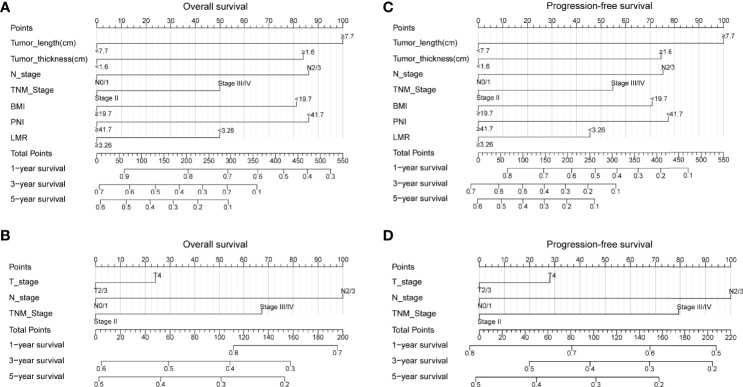
Nomogram for predicting the probability of 1-, 3-, and 5-year OS and PFS for the whole study population. **(A)** A nomogram that integrates tumor length, tumor thickness, N stage, TNM stage, BMI, PNI, and LMR for OS in ESCC patients; **(B)** A nomogram that integrates T stage, N stage, and TNM stage for OS in ESCC patients; **(C)** A nomogram that integrates tumor length, tumor thickness, N stage, TNM stage, BMI, PNI, and LMR for PFS in ESCC patients; **(D)** A nomogram that integrates T stage, N stage, and TNM stage for PFS in ESCC patients. OS, overall survival; PFS, progression-free survival; T, tumor; N, node; TNM, tumor-node-metastasis; BMI, body mass index; PNI, prognostic nutrition index; LMR, lymphocyte-to-monocyte ratio; ESCC, esophageal squamous cell carcinoma.

The above results suggested that tumor length, tumor thickness, N stage, TNM stage, BMI, PNI, and LMR were the independent prognostic factors for ESCC. Therefore, we established prediction models for OS and PFS by fitting these clinicopathological parameters. A higher nomogram score represented a worse prognostic factor. The calibration curve was used to evaluate the performance of the nomogram. The discriminative ability of the nomogram models was compared with that of the 8th AJCC TNM staging. The C-index of 1-, 3-, and 5-year for OS in our prediction model were 0.710 (CI, 0.667–0.752), 0.716 (CI, 0.679–0.753), and 0.764 (CI, 0.723–0.806), respectively. The C-index of 1-, 3-, and 5-year for OS in the 8th TNM staging criteria were 0.611 (CI, 0.569–0.653), 0.637 (CI, 0.601–0.674), and 0.664(CI, 0.620–0.708), respectively. The C-index of 1-, 3-, and 5-year for PFS in our prediction model were 0.700 (CI, 0.665–0.736), 0.730 (CI, 0.694–0.766), and 0.761 (CI, 0.719–0.803), respectively. The C-index of 1-, 3-, and 5-year for PFS in the 8th TNM staging criteria were 0.618 (CI, 0.580–0.656), 0.646 (CI, 0.606–0.686), and 0.680(CI, 0.635–0.727), respectively. Our prediction model was superior to the 8th TNM staging criteria in the prediction of 1-, 3-, and 5-year OS and PFS by using the Delong’s test (p < 0.001 for all) (**Figure 1**). The calibration curve for predicting the probability of 1-, 3-, and 5-year OS and PFS of the nomogram was shown in **Supplementary Figure 1**. In summary, our novel nomogram models may be better models for predicting the survival of patients with ESCC who received dCRT than the 8th TNM staging criteria alone.

### The Relationship Between the Tumor Length, Tumor Thickness, and Survival

**Figure 2:**
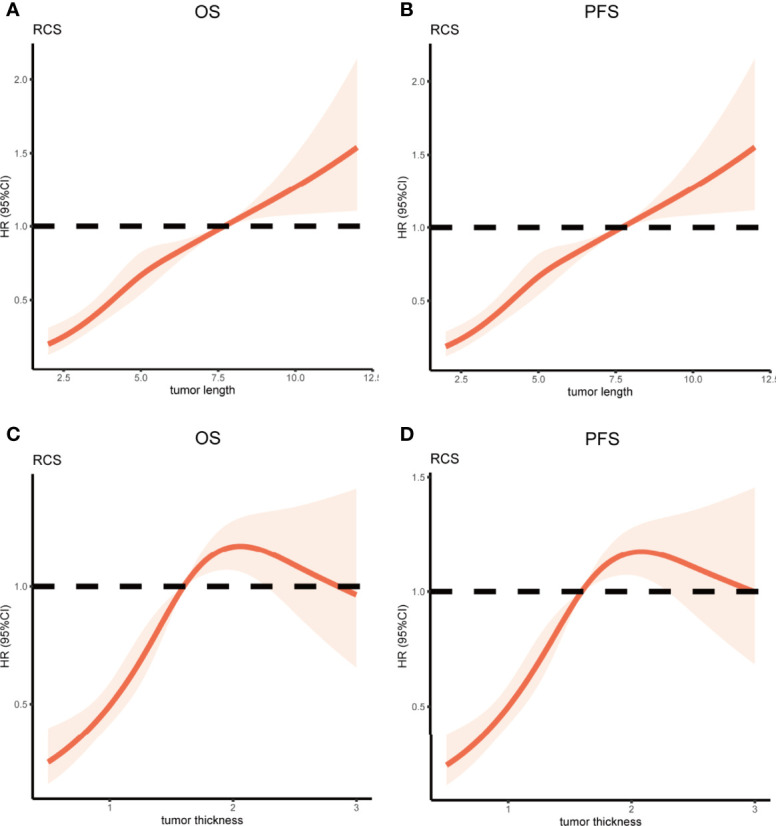
Restricted Cubic Spline analysis was used to classify the association between tumor length, tumor thickness, and survival in ESCC patients. The hazard ratio derived from a Multivariate Cox model is shown on the y-axis. The 95% CI of the adjusted hazard ratio are represented by the shaded area. The 7.7cm and 1.6cm are the reference of tumor length and tumor thickness (HR=1). **(A, B)** A nonlinear relationship between the tumor length and survival for patients with ESCC. The death hazard of tumor length sharply increased at 7.7cm (p < 0.001 for non-linearity); **(C, D)** A nonlinear relationship between the tumor thickness and survival for patients with ESCC. The death hazard of tumor thickness sharply increased at 1.6cm (p < 0.001 for non-linearity). ESCC, esophageal squamous cell carcinoma; HR, hazard ratio; CI, confidence interval; OS, overall survival; PFS, progression-free survival.

Restricted cubic spline (RCS) analysis was used to classify the association between tumor length, tumor thickness, and survival. **Figures 2A, B** demonstrated a nonlinear relationship between the tumor length and OS as well as PFS for patients with ESCC. The death hazard of tumor length sharply increased at 7.7cm (p < 0.001 for non-linearity). The results also demonstrated a nonlinear relationship between the tumor thickness and survival. The death hazard of tumor thickness sharply increased at 1.6cm (p < 0.001 for non-linearity, **Figures 2C, D**).

### Subgroup Analyses of the Relationship Between PI and Outcome

**Figure 3:**
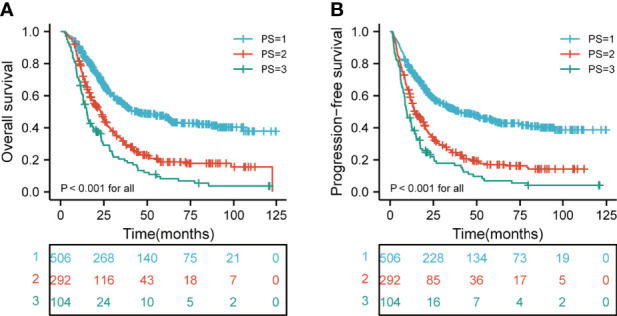
Kaplan-Meier curves according to different risk groups for the whole study population showing **(A)** Risk stratification for PI on OS (p < 0.001 for all); **(B)** Risk stratification for PI on PFS (p < 0.001 for all). PI, prognostic index; OS, overall survival; PFS, progression-free survival.

We next investigated the association between the PI category and survival. The median OS was 25.9 months (range, 2.6-124.7), 20.2 months (range, 2.1-122.8), and 15.7 months (range, 2.8-121) for the low-risk, medium-risk, and high-risk groups, respectively. The 3- OS rates in the low-risk, medium-risk, and high-risk groups were 55.2%, 31.7%, and 20.6%, respectively. The 5-year OS rates in the low-risk, medium-risk, and high-risk groups were 47.1%, 18.7%, and 8.2%, respectively. In addition, the median PFS was 21.6 months (range, 1.6-124.7), 13.8 months (range, 1.1-112.1), and 9.7 months (range, 1.2-121) for the low-risk, medium-risk, and high-risk groups, respectively. The 3- PFS rates in the low-risk, medium-risk, and high-risk groups were 51.9%, 24.6%, and 17.9%, respectively. The 5-year PFS rates in the low-risk, medium-risk, and high-risk groups were 44.2%, 17.0%, and 6.9%, respectively. In stratified analysis, as shown in **Figures 3A, B**, the higher risk group was significantly associated with shorter OS and PFS in ESCC patients (both p < 0.001 for all).

### Kaplan-Meier Curves of Different Risk Groups According to the Clinical T Stage, N Stage, and TNM Stage

**Figure 4:**
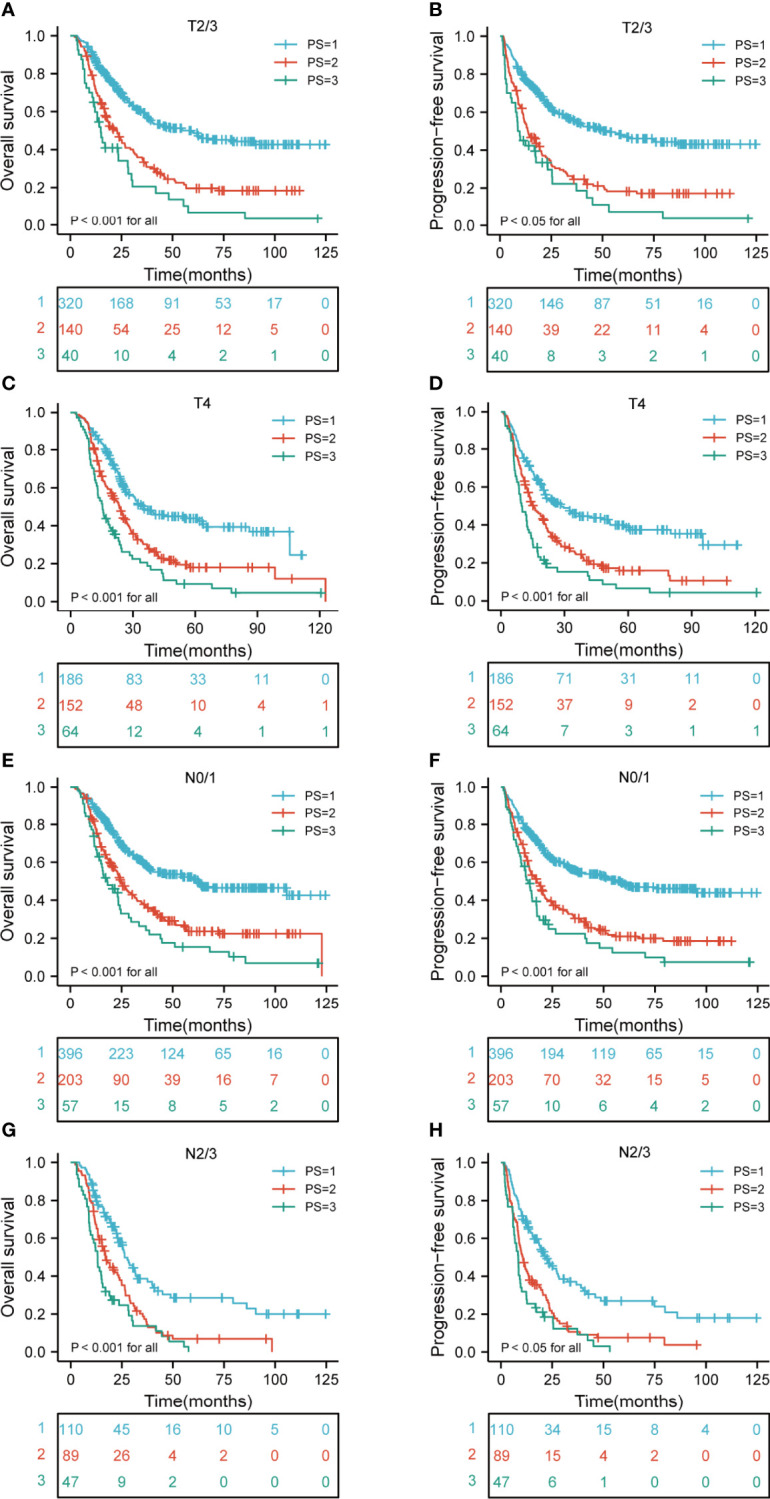
Kaplan-Meier curves according to T and N stage categories for the whole study population according to risk groups showing **(A–D)** OS and PFS (p < 0.05 for all) of patients with T2-4; **(E–H)** OS and PFS (p < 0.001 for all) of patients with N0-3. T, tumor; N, node; OS, overall survival; PFS, progression-free survival.

**Figure 5:**
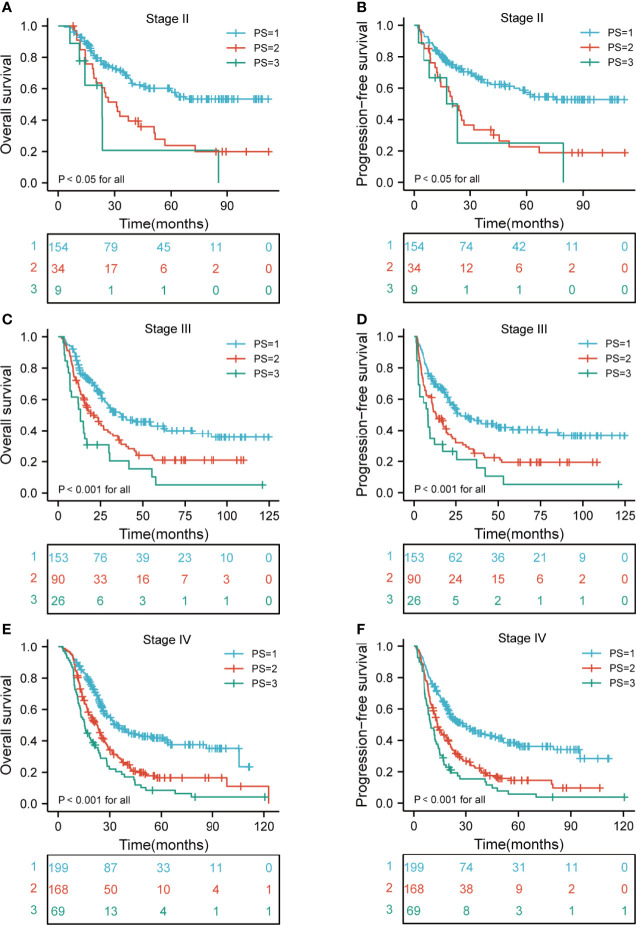
Kaplan-Meier curves according to TNM stage categories for the whole study population according to risk groups showing **(A–F)** OS and PFS (p < 0.05 for all) of patients with stage II-IVA. TNM, tumor-node-metastasis; OS, overall survival; PFS, progression-free survival.

A comparison of the OS rates in different risk groups showed that there were significant differences for ESCC patients with clinical T2-4 and N0-3 stage groups (all p < 0.05). Similarly, there were significant differences in PFS for ESCC patients with clinical T2-4 and N0-3 stage groups (all p < 0.05) (**Figures 4A–H**). We also carried out the analysis between different risk groups according to the clinical TNM stage. The results revealed that there were significant differences for ESCC patients with clinical II-IVA stage groups of the OS rates in different risk groups (all p < 0.05). Interestingly, there were significant differences in PFS for ESCC patients with clinical II-IVA stage groups (all p < 0.05) (**Figures 5A–F**).

## Discussion

Although much progress has been made in radical surgery, chemotherapy, and radiotherapy, the survival rate of patients with ESCC remains low. Recurrence occurs even in patients with ESCC who received dCRT. Hence, identifying an easily available prognostic model for ESCC is vital for clinicians to develop appropriate treatment plans. The current TNM staging system is the standard classification system for predicting the prognosis of patients (18). However, it is not accurate for predicting the prognosis of patients with EC. It is also not suitable for patients receiving dCRT (19). An increasing number of researchers are focusing on the non-operative staging of EC (20). In our study, we found a new better model to predict the prognosis of patients with ESCC who received dCRT than the TNM classification, which will help clinicians to evaluate the patient prognosis after treatment. At the same time, it is beneficial for selecting the optimal clinical treatment and ultimately improving the patient prognosis.

Despite several studies have shown that tumor length and tumor thickness are associated with the prognosis of EC, they have only analyzed the association between one of these indicators and prognosis. To the best of our knowledge, this is the first study to evaluate the clinical prognostic significance of combining the tumor length, tumor thickness, N stage, TNM stage, BMI, PNI, and LMR in ESCC patients received dCRT. In addition, it is also the first study to compare the TNM classification and other models on the prognostic evaluation of ESCC treated with dCRT.

Growing evidence has revealed a relationship between tumor length and prognosis of EC (8, 9, 21, 22), which is consistent with our results. Esophageal tumor thickness is also an essential factor affecting the prognosis of EC. Our study results showed that the optimum cutoff values for tumor length and thickness were 7.7 cm and 1.6 cm, respectively. Restricted cubic spline (RCS) analysis demonstrated a nonlinear relationship between the tumor length, tumor thickness, and survival for patients with ESCC. The death hazard of tumor length and tumor thickness sharply increased at 7.7 cm and 1.6 cm. Although most studies have suggested that tumor size is an important prognostic factor for ESCC, there is no consensus on the prognostic cutoff value for tumor size. Recent studies have shown that the cutoff value for tumor length is 2-6 cm (7–9, 21), The cutoff values were different owing to the heterogeneity between the studies. The difference in the sample size is also an important reason for the inconsistent research results. In the present study, we confirmed the independent prognostic value of tumor length and thickness, which suggests that these two indicators should be considered when establishing the non-operative staging of ESCC.

In recent years, several studies have shown that the systemic inflammatory response (SIR) is an important prognostic indicator. There have been a number of studies on various prognostic biomarkers of SIR. The relationship between these parameters and the prognosis of patients can be explained by the interaction between tumor immune/inflammatory cells and the surrounding normal tissues, which are momentous for cancer occurrence and development. Previous studies have indicated that substances such as TNF-α and IL-6, which are produced by tumors, may affect the inflammatory markers (23). In theory, directly measuring the level of these indices in the serum is the best method to estimate the changes in the inflammatory indices caused by the interaction between the tumor and host tissues. However, routine testing of these indicators among cancer patients is expensive and extremely inconvenient. Therefore, it is reasonable to identify alternative suitable biomarkers. Inflammatory indices are easy to obtain and low-cost indicators of systemic inflammation, and have been studied as prognostic markers for several solid organ tumors (24).

Our results showed that in univariate analysis, LMR, NLR, and PLR were the significant factors affecting the prognosis of patients with ESCC. Interestingly, in multivariate analysis, only LMR was an independent risk factor. LMR is an integral part of the SIR. It has been widely studied as a predictor or prognostic factor for advanced cancers, including ESCC, gastric cancer, ovarian cancer, and oral cancer (25–28). With regard to defining the threshold for elevated LMR, previous studies applied critical values from 2.95 to 4 in patients with EC (25, 29, 30). Similarly, we defined the threshold for PLR elevation as 3.26, which is consistent with the threshold of previous studies. The difference of cutoff value can be explained by the different sample sizes and clinicopathological features examined in each study, as well as the different statistical methods used for calculating the best cutoff values. In the long run, the combined measurement of lymphocyte and monocyte may provide more prognostic information than any single component alone. Our study results show that it is a useful baseline variable that can be used to evaluate the prognosis of patients with ESCC who are considering dCRT treatment.

Clinical N stage is a crucial parameter in treatment decisions and prognosis in patients with EC. It is vital in delineation of radiation tumor volumes in EC. Estimate of the N stage before dCRT is momentous for predicting the prognosis and for planning the treatment strategy. Since there is no clear detection method to provide staging information about the status of lymph nodes, clinicians usually use CT, EUS, and PET-CT in combination to minimize the risk of missed diagnosis. In our study, N stage was an independent prognostic factors associated with survival. It further showed that N stage has an important effect on the prognosis of patients with EC. Based on the results of multivariate analysis, we established a nomogram that integrates tumor length, tumor thickness, N stage, TNM stage, BMI, PNI, and LMR in ESCC patients. Our prediction models had higher C-index for OS and PFS, which might be superior to that of the 8th TNM staging criteria. Therefore, our novel nomogram models may be better models for predicting the survival of patients with ESCC who received dCRT than the 8th TNM staging criteria alone. This nomogram’s primary significance is that it can predict the prognosis of non-operative ESCC patients, and help clinicians and patients to make appropriate decisions about treatment plans. For example, it is often difficult to quantify the potential benefits and actual risks of a given treatment in ESCC patients who are suitable for dCRT. Making such decisions based on the new prognostic model might help individual patients to weigh whether the side effects of dCRT are worth the risk, especially if their prognosis is poor based on the pre-treatment prognostic scores. Considering the differences in the survival rates, this new prognostic model may be considered before selecting dCRT.

Our study has several limitations. First, this was a retrospective study, and the results are limited. Therefore, the results need to be confirmed by further prospective studies to reach better conclusions. Second, our study is limited to patients with ESCC and has no guiding significance for patients with other types of EC. Third, the study’s sample size was insufficient, and subsequent studies with more samples are needed to confirm our results. In addition, the follow-up period ranged too long for OS and PFS, which may cause biased analysis. Finally, other confounding factors that might influence the values of inflammatory indices, our proposed cutoff values should be verified by other institutions.

## Conclusion

In summary, we demonstrated that tumor length and tumor thickness are independent predictors of poor prognosis in patients with ESCC undergoing dCRT. Our prediction models might be superior to that of the 8th TNM staging criteria. The tumor length and tumor thickness should be considered as prognostic factors for esophageal squamous cell carcinoma. The study results suggest that the new models integrating these factors may provide a simple and widely available method for evaluating the prognosis of non-operative ESCC patients, but further large-scale studies with standard assessing and well-designed methods are warranted to confirm the present findings in the future.

## Data Availability Statement

The data that support the findings of this study are available from the corresponding author upon reasonable request.

## Ethics Statement

The current study was approved by the ethics committee of Fujian Medical University Cancer Hospital (YKT2021-005-01), Fuzhou, China and conducted in accordance with the principles of the Declaration of Helsinki and its amendment. All patients provided written informed consent prior to treatment, and all the information was anonymized prior to analysis.

## Author Contributions

WC, QY, JL, XC, and HW designed this study. YY, TL, QZ, HL, YW, and ZW contributed to the data collection. XC, YY, JQ, DK, and YW analyzed the data. WC, XC, JL, HW, and QY supervised the study. YY, HZ, JQ, ML, JY, and LL wrote the manuscript. All authors reviewed and approved the final manuscript.

## Funding

This work was supported by the Joint Funds for the innovation of Science and Technology, Fujian province (2018Y9111, 2019Y9041, 2020Y9036), the Joint Funds for the Financial Foundation of Fujian Province ((2020)729), the grants of Fujian Provincial Clinical Research Center for Cancer Radiotherapy and Immunotherapy (2020Y2012), the National Clinical Key Specialty Construction Program (2021), and the National Natural Science Foundation of China (81803037).

## Conflict of Interest

The authors declare that the research was conducted in the absence of any commercial or financial relationships that could be construed as a potential conflict of interest.

## Publisher’s Note

All claims expressed in this article are solely those of the authors and do not necessarily represent those of their affiliated organizations, or those of the publisher, the editors and the reviewers. Any product that may be evaluated in this article, or claim that may be made by its manufacturer, is not guaranteed or endorsed by the publisher.

## Supplementary Material

Supplementary Figure 1 Calibration curve for predicting the probability of 1-, 3-, and 5-year OS and PFS for the whole study population. **(A–C)** The calibration curve for predicting the probability of 1-, 3-, and 5-year OS of the novel nomogram; **(D–F)** The calibration curve for predicting the probability of 1-, 3-, and 5-year OS of the TNM staging nomogram; **(G–I)** The calibration curve for predicting the probability of 1-, 3-, and 5-year PFS of the novel nomogram; **(J–L)** The calibration curve for predicting the probability of 1-, 3-, and 5-year PFS of the TNM staging nomogram. OS, overall survival; PFS, progression-free survival; TNM, tumor-node-metastasis.

## Abbreviations

ESCC, esophageal squamous cell carcinoma; dCRT, definitive chemoradiotherapy; OS, overall survival; PFS, progression-free survival; RCS, Restricted Cubic Splines; PI, prognostic index; EC, Esophageal carcinoma; EAD, esophageal adenocarcinoma; T, tumor; N, node; M, metastasis; TNM, tumor-node-metastasis; CT, computed tomography; IMRT, intensity modulated radiotherapy; 3D-CRT, three dimensional-conformal radiotherapy; EUS, endoscopic ultrasound; PET-CT, positron emission tomography-CT; MLD, mean lung dose; GTV, gross tumor volume; CTV, clinical target volume; PTV, planning target volume; 5-FU, 5-fluorouracil; BMI, body mass index; PNI, prognostic nutrition index; LMR, lymphocyte-to-monocyte ratio; NLR, neutrophil-to-lymphocyte ratio; PLR, platelet-to-lymphocyte ratio; SIR, systemic inflammatory response; HR, hazard ratio; CI, confidence interval.
