# Supplementary figures and images for: A Novel Model Combining Tumor Length, Tumor Thickness, TNM_Stage, Nutritional Index, and Inflammatory Index Might Be Superior to the 8th TNM Staging Criteria in Predicting the Prognosis of Esophageal Squamous Cell Carcinoma Patients Treated With Definitive Chemoradiotherapy
Source: Front Oncol. 2022 Jun 1;12:896788. doi: 10.3389/fonc.2022.896788 (PMC9198351; doi:10.3389/fonc.2022.896788)

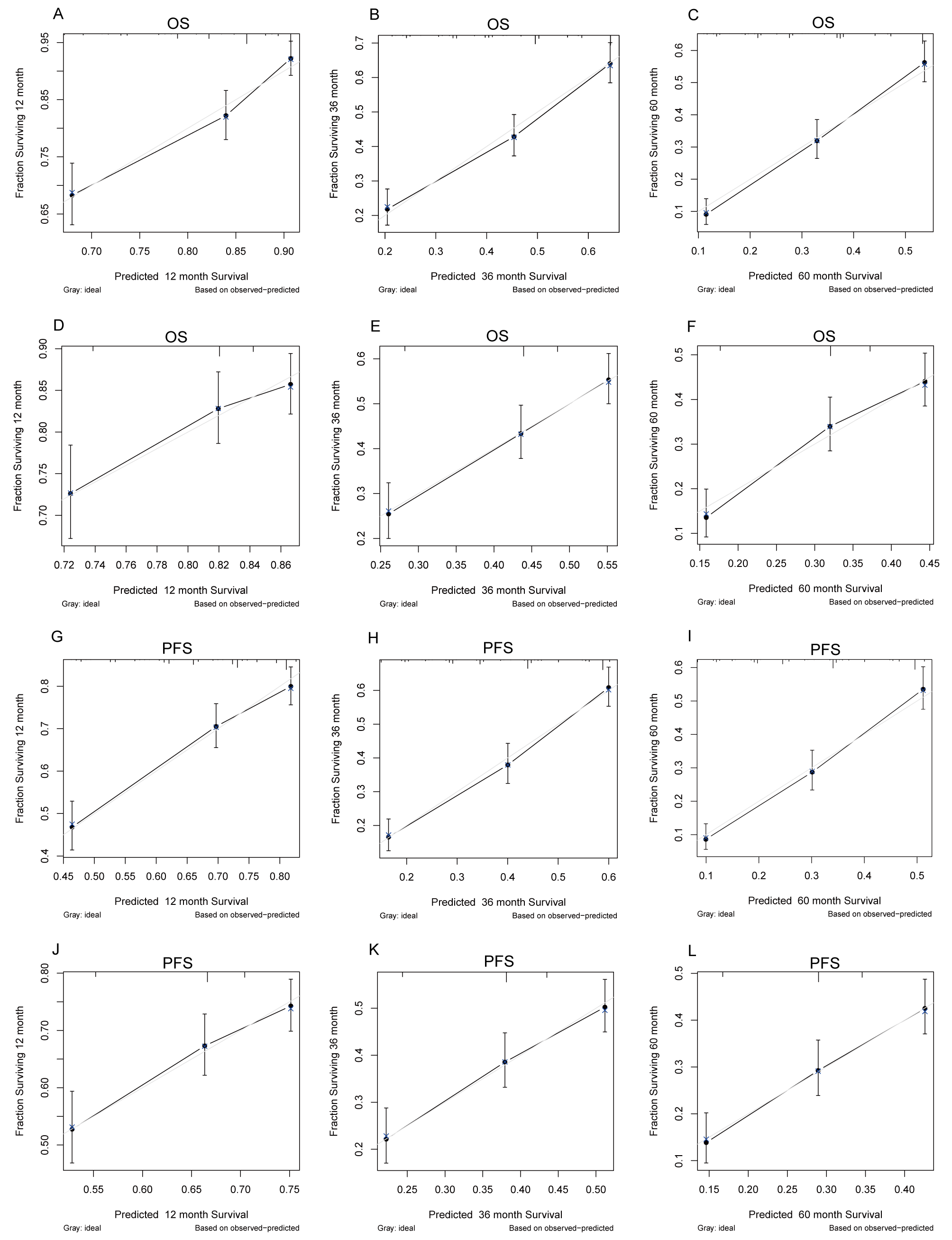

Supplement: Supplementary Figure 1 — Calibration curve for predicting the probability of 1-, 3-, and 5-year OS and PFS for the whole study population. (A–C) The calibration curve for predicting the probability of 1-, 3-, and 5-year OS of the novel nomogram; (D–F) The calibration curve for predicting the probability of 1-, 3-, and 5-year OS of the TNM staging nomogram; (G–I) The calibration curve for predicting the probability of 1-, 3-, and 5-year PFS of the novel nomogram; (J–L) The calibration curve for predicting the probability of 1-, 3-, and 5-year PFS of the TNM staging nomogram. OS, overall survival; PFS, progression-free survival; TNM, tumor-node-metastasis. [file Image_1.tif]
